# Supraclavicular and/or celiac lymph node metastases from thoracic esophageal squamous cell carcinoma did not compromise survival following neoadjuvant chemoradiotherapy and surgery

**DOI:** 10.18632/oncotarget.12200

**Published:** 2016-09-22

**Authors:** Won Kyung Cho, Dongryul Oh, Yong Chan Ahn, Young Mog Shim, Jae Ill Zo, Jong-Mu Sun, Myung-Ju Ahn, Keunchil Park

**Affiliations:** ^1^ Department of Radiation Oncology, Samsung Medical Center, Sungkyunkwan University School of Medicine, Gangnam-gu, Seoul, Korea; ^2^ Department of Thoracic and Cardiovascular Surgery, Samsung Medical Center, Sungkyunkwan University School of Medicine, Gangnam-gu, Seoul, Korea; ^3^ Medicine (Division of Hematology-Oncology), Samsung Medical Center, Sungkyunkwan University School of Medicine, Gangnam-gu, Seoul, Korea

**Keywords:** esophageal cancer, neoadjuvant chemoradiotherapy, supraclavicular lymph node, celiac lymph node, staging

## Abstract

This study is to evaluate the prognostic significance of supraclavicular and/or celiac lymph node (LN) metastases in locally advanced thoracic esophageal squamous cell carcinoma (ESCC) patients treated with neoadjuvant chemoradiotherapy (NACRT) and surgery. Among the total 199 patients, 75 (37.7%) had supraclavicular and/or celiac LN metastasis. Surgery was performed following NACRT in 168 patients (84.4%). After the median 18.7 (1.0-147.2) months’ follow-up, 2-year rates of progression-free survival (PFS) and overall survival (OS) in all patients were 48.1% and 65.7%, respectively. In multivariate analyses, negative surgical margin (*p* < 0.001), ypT0 stage (*p* = 0.004), and ypN0 stage (*p* = 0.020) were significantly favorable factors for PFS, and negative surgical margin (*p* < 0.001) was the only significantly favorable factor for OS. Metastasis to the supraclavicular and/or celiac LNs was significant factor neither for PFS (*p* = 0.311) nor OS (*p* = 0.515). Supraclavicular and/or celiac LN metastasis did not compromise the clinical outcomes following NACRT and surgery.

## INTRODUCTION

The prognosis of resectable esophageal cancer remains still unsatisfactory with 5-year overall survival (OS) rates of 15%~34% [1, 2]. A multicenter randomized trial demonstrated that the addition of neoadjuvant chemoradiotherapy (NACRT) and surgery improved OS at 5 years when compared to surgical resection alone in patients with resectable stage (T2-3N0-1M0) (47% *vs*. 34%, *p* = 0.003) [3]. Several meta-analyses also concluded that NACRT and surgery showed benefit when compared to surgery with or without neoadjuvant chemotherapy in locally advanced esophageal cancer [4, 5]. Thus, NACRT and surgery has been now recommended as the standard treatment in resectable locally advanced esophageal cancer.

Esophageal cancer can spread bidirectionally through the lymphatics to reach remote locations ranging from the cervical to abdominal lymph nodes (LNs). The 6^th^ edition of American Joint Committee on Cancer (AJCC) staging system [6] defined the regional and non-regional LNs based on the primary tumor location: for thoracic esophageal cancer, upper periesophageal, subcarinal, and lower periesophageal LNs were regional LNs; while cervical and celiac LNs were non-regional LNs. Cervical LN metastasis from upper thoracic esophageal cancer and celiac LN metastasis from lower thoracic esophageal cancer were classified as M1a stage considering the distance between the primary tumor and metastatic LNs, however, metastases to more distantly located LNs were classified as M1b together with distant organ metastasis. The 7^th^ edition of AJCC staging system re-defined the regional LNs as any periesophageal LN from cervical to celiac LN and removed M1a and M1b classifications, but incorporated the numbers of metastatic LN instead [7].

The previous clinical trials, which established NACRT and surgery as the current standard for locally advanced esophageal cancer, mostly included the patients with cM0, but not cM1a/b, stage according to the 6^th^ edition of AJCC staging system. The current study aimed to evaluate the prognostic influence of cM1a/b by the 6^th^ AJCC system in patients with locally advanced thoracic esophageal squamous cell carcinoma following NACRT and surgery.

## RESULTS

### Patient characteristics

The patients’ characteristics are summarized in Table 1. The median age of all patients was 62 (37~80) years and the majority of patients were male (190/199, 95.5%). The most frequent location of the primary tumor was the upper thoracic esophagus in 92 patients (46.2%), followed by the middle thoracic in 62 (31.2%) and lower thoracic in 45 (22.6%), respectively. Most patients had good to excellent Eastern Cooperative Oncology Group (ECOG) performance status of 0-1 (194/199, 97.5%). Clinical T stages were cT1 in six patients (3.0%), cT2 in 39 (19.6%), cT3 in 149 (74.9%), and cT4 in five (2.5%), respectively, and majority had LN metastasis (188/199, 94.5%). Had the 6^th^ edition AJCC staging system been applied, 54 (27.1%) and 21 (10.6%) patients would have been classified as cM1a and cM1b stages, respectively. Among the M1a patients, 42 patients had upper thoracic esophageal cancer with supraclavicular LN metastasis and 12 had lower thoracic esophageal cancer with celiac LN metastasis.

**Table 1:** Patients’ characteristics (*N* = 199)

Characteristics	Number of patients
Gender	
Male	190 (95.5%)
Female	9 (4.5%)
Median age (range)	62 (37~80) years
ECOG performance status	
0	12 (6%)
1	182 (91.5%)
2	4 (2.0%)
3	1 (0.5%)
Endoscopic ultrasonography done	116 (58.3%)
Tumor location	
Upper thoracic	92 (46.2%)
Middle thoracic	62 (31.2%)
Lower thoracic	45 (22.6%)
cT stage	
cT1	6 (3.0%)
cT2	39 (19.6%)
cT3	149 (74.9%)
cT4	5 (2.5%)
cN stage	
cN0	11 (5.5%)
cN+	188 (94.5%)
cM stage according to the 6^th^ AJCC system	
cM0	124 (62.3%)
cM1a	54 (27.1%)
cM1b	21 (10.5%)

Abbreviations: ECOG, Eastern Cooperative Oncology Group; AJCC, American Joint Committee on Cancer

### Treatment compliance

Compliance to NACRT was very high: 196 patients (98.5%) could complete the planned radiotherapy (RT) dose (median RT dose = 44 (3.6~45) Gy); and 191 (96.0%) could complete the 2 cycles of chemotherapy. Surgery was performed in 168 patients (84.4%) and the reasons for not undergoing surgery were patients’ refusal of surgery in 14 (7.0%), inadequate recovery from NACRT toxicity in six (3.0%), identification of disease progression before surgery in five (2.5%), development of esophago-respiratory fistula during NACRT in three (1.5%), and death while waiting for surgery in three (1.5%). Among 14 patients who refused surgery, seven received further chemoradiotherapy, while the remaining seven declined any further treatment. Five patients who developed disease progression following NACRT were recommended to receive palliative systemic chemotherapy. Three patients died while waiting for surgery and were assumed to have experienced esophageal perforation and subsequent massive bleeding. The median interval between NACRT completion and surgery was 5.0 (1.6-13.6) weeks. Two- and three-field LN dissections were performed in 79 and 89 patients (47.0% and 53.0%), respectively. Post-surgical mortality occurred in eleven patients (6.5%): pulmonary complication in nine; anastomosis leakage in one; and tracheoesophageal fistula in one, respectively.

### Pathologic assessment

R0 resection was achieved in 153 patients (91.1%) (Table 2). Pathologic complete response of primary tumor (ypT0) was achieved in 77 patients (45.8%). The median number of LNs retrieved was 38 (3-86) and 75 patients (44.6%) had ypN0 stage. Overall, 44 patients (26.2%) achieved pathologic complete response both in the primary tumor and LNs (pCR, ypT0N0).

**Table 2:** Lymph node dissection and pathologic assessment following surgery (*N* = 168)

Characteristics	Number of patients
Surgical procedure	
2 field lymph node dissection	79 (47.0%)
3 field lymph node dissection	89 (53.0%)
Median number of lymph node dissected	38 (3~86)
Surgical margin	
Negative	153 (91.1%)
Microscopically positive	8 (4.8%)
Gross residual	7 (4.2%)
ypT stage	
ypT0	77 (45.8%)
ypT1	23 (13.7%)
ypT2	24 (14.3%)
ypT3	42 (25.0%)
ypT4	2 (1.2%)
ypN stage	
ypN0	75 (44.6%)
ypN1	56 (33.3%)
ypN2	23 (13.7%)
ypN3	14 (8.3%)
Pathologic complete response	
Yes	44 (26.2%)
No	124 (73.8%)

### Clinical outcomes

**Figure 1:**
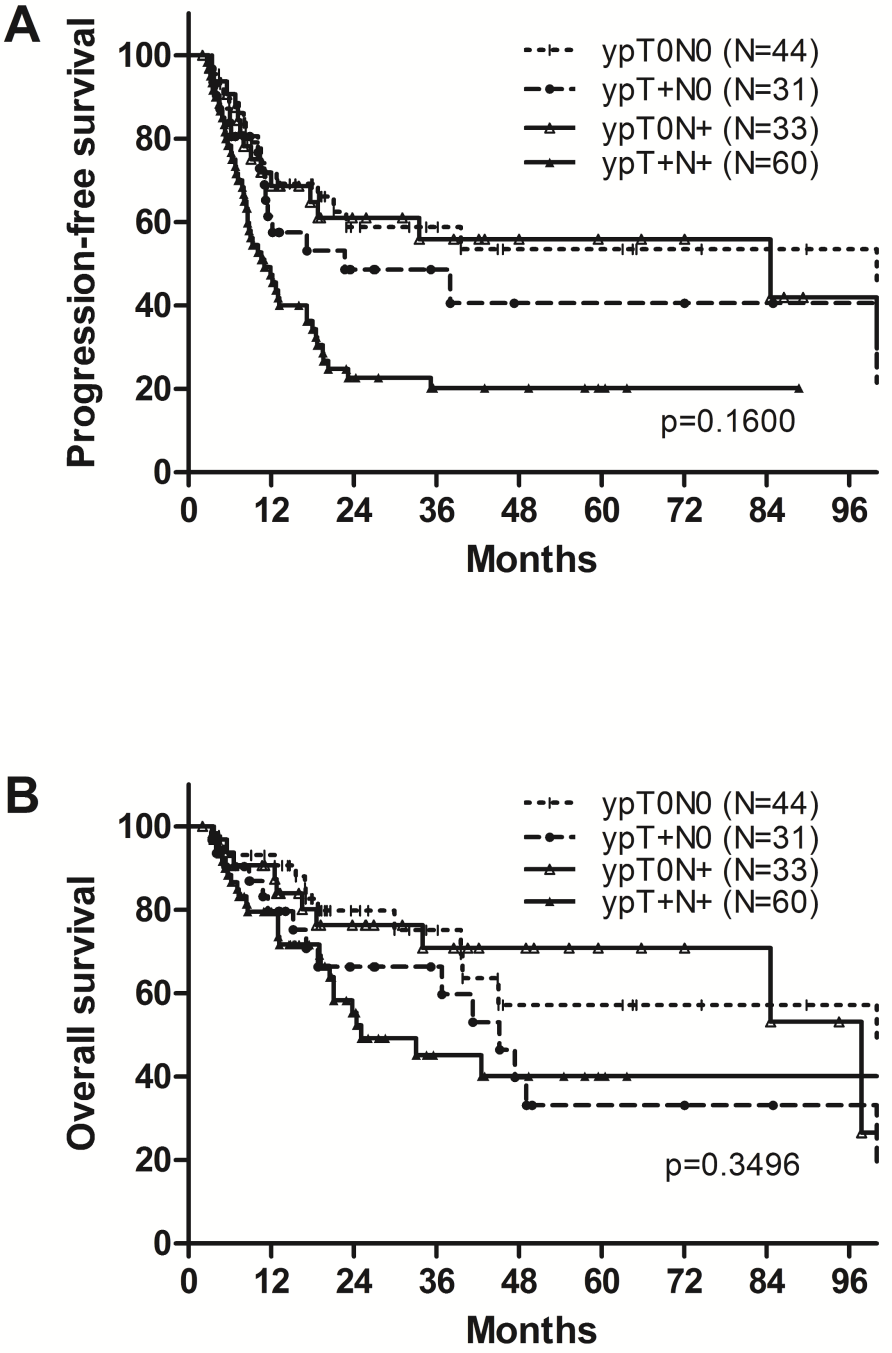
A. Progression-free survival and B. overall survival of all patients

**Figure 2:**
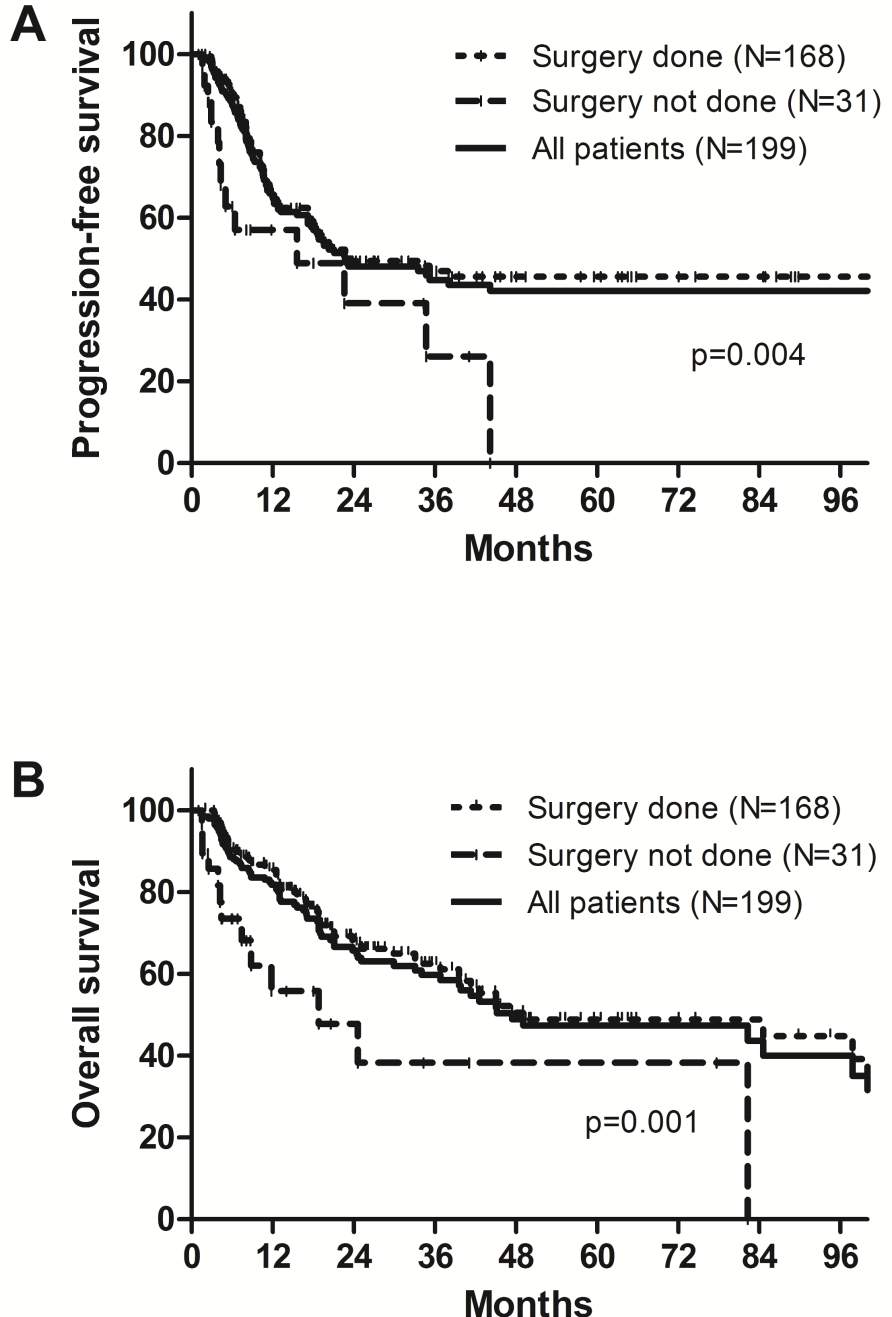
A. Progression-free survival and B. overall survival according to ypT and ypN stages

After the median 18.7 (1.0-147.2) months’ follow-up on 199 patients, 88 (44.2%) experienced disease progression and 77 (38.7%) died. The patterns of failure were LR failure in 33 patients (37.5%), distant metastasis in 32 (36.4%), and combined LR and distant failure in 23 (26.1%), respectively. The sites of LR failure in relation to the RT volume were analyzed. Among 56 patients who developed LR failure, 27 patients experienced recurrence inside the radiation volume, 26 did outside the radiation volume, and three did both inside and outside the radiation volume, respectively. The 2-year rates of progression-free survival (PFS) and OS of all patients were 48.1% and 65.7%, respectively. The clinical outcomes at 2 years of the patients who underwent surgery were significantly better than those who did not: PFS rates were 49.4% and 37.3% (*p* = 0.004); and OS rates were 68.2% and 45.8% (*p* = 0.001), respectively (Figure 1). Among 168 patients who underwent surgery, 2-year rates of PFS and OS based on yp-stages were 58.8% and 79.8% in ypT0N0, 61.0% and 76.3% in ypT0N+, 48.7% and 66.3% in ypT+N0, and 22.7% and 55.4% in ypT+N+, respectively (Figure 2).

The probable prognosticators were analyzed by the univariate analyses in 168 patients who underwent surgery. The factors included gender, age, primary tumor location, cT stage, cN stage, 6^th^ AJCC M stage, surgical procedure, ypT stage, ypN stage, pCR, adjuvant chemotherapy, and surgical margin (Table 3). The significantly favorable factors on loco-regional control (LRC) at 2 years were negative surgical margin (*p* < 0.001), ypT0 (*p* = 0.001), cT1-2 (*p* = 0.040), pCR (*p* = 0.007), and ypN0 (*p* = 0.021). Favorable factors on PFS at 2 years were negative surgical margin (*p* < 0.001), ypT0 (*p* = 0.001), pCR (*p* = 0.014), and ypN0 (*p* = 0.021), and those on OS were negative surgical margin (*p* < 0.001), ypT0 (*p* = 0.011), and age of 60 years or younger (*p* = 0.017). In the multivariate analysis, negative surgical margin (HR 0.263; 95% CI 0.132-0.522; *p* < 0.001), ypT0 stage (HR, 0.413; 95% CI 0.228-0.748; *p* = 0.004), and ypN0 stage (HR 0.490; 95% CI 0.269-0.892; *p* = 0.020) were statistically significant favorable prognostic factors for PFS at 2 years. Negative surgical margin (HR 0.187; 95% CI 0.086-0.404; *p* < 0.001) was the only favorable factor for OS at 2 years (Table 4).

**Table 3:** Prognostic factors by univariate analyses (*N* = 168)

Factors	2yr-LRC	*p*	2yr-PFS	*p*	2yr-OS	*p*
Gender		*0.643*		*0.778*		*0.280*
Male (*N* = 159)	60.8%		43.8%		69.0%	
Female (*N* = 9)	57.1%		44.4%		55.6%	
Age		*0.122*		*0.699*		***0.017***
≤60 years (*N* = 76)	51.7%		41.0%		76.4%	
>60 years (*N* = 92)	69.7%		46.4%		61.1%	
Tumor location		*0.454*		*0.131*		*0.121*
Upper thoracic (*N* = 76)	64.0%		46.3%		69.2%	
Middle thoracic (*N* = 52)	58.6%		34.0%		61.2%	
Lower thoracic (*N* = 40)	55.8%		51.2%		76.2%	
cT stage		***0.040***		*0.792*		*0.532*
cT1-2 (*N* = 38)	47.0%		43.4%		66.0%	
cT3-4 (*N* = 130)	65.8%		43.9%		68.8%	
cN stage		*0.868*		*0.630*		*0.592*
cN0 (*N* = 9)	63.5%		55.6%		55.6%	
cN+ (*N* = 159)	60.4%		43.0%		69.0%	
cM stage (6^th^ AJCC system)		*0.481*		*0.311*		*0.515*
cM0 (*N* = 104)	64.0%		49.5%		69.3%	
cM1a/b (*N* = 64)	56.0%		36.3%		67.4%	
Lymph node dissection		*0.074*		*0.486*		*0.890*
2 fields (*N* = 79)	53.1%		40.9%		67.8%	
3 fields (*N* = 89)	67.0%		46.1%		68.8%	
ypT stage		***0.001***		***0.001***		***0.011***
ypT0 (*N* = 77)	77.5%		60.1%		78.3%	
ypT1-4 (*N* = 91)	45.4%		30.4%		59.6%	
ypN stage		***0.021***		***0.021***		*0.472*
ypN0 (*N* = 75)	71.4%		54.1%		74.4%	
ypN+ (*N* = 93)	51.7%		35.8%		63.4%	
p-complete response (pCR)		***0.007***		***0.014***		*0.118*
pCR (*N* = 44)	80.8%		58.8%		79.8%	
Non-pCR (*N* = 124)	53.3%		38.4%		64.3%	
Adjuvant chemotherapy		*0.374*		*0.310*		*0.104*
Yes (*N* = 36)	64.3%		48.2%		73.4%	
No (*N* = 132)	52.5%		42.7%		66.7%	
Surgical margin		***<0.001***		***<0.001***		***<0.001***
Negative (*N* = 153)	63.1%		48.1%		72.7%	
Positive (*N* = 15)	21.5%		0		19.0%	

Abbreviations: LRC, locoregional control; PFS, progression free survival; OS, overall survival

**Table 4:** Prognostic factors by multivariate analyses (*N* = 168)

Factors	Locoregional control		Progression-free survival		Overall survival	
	HR (95% CI)	*p*	HR (95% CI)	*p*	HR (95% CI)	*p*
Gender	0.863	*0.832*	0.852	*0.751*	0.493	*0.215*
Male vs. female	(0.221-3.370)		(0.316-2.294)		(0.161-1.508)	
Age	1.496	*0.198*	1.086	*0.708*	0.582	*0.582*
≤60 years vs. >60 years	(0.810-2.762)		(0.706-1.671)		(0.333-1.019)	
Location						
Upper vs. lower	1.016	*0.973*	1.132	*0.734*	1.24	*0.65*
	(0.403-2.559)		(0.554-2.314)		(0.489-3.144)	
Middle vs. lower	1.316	*0.506*	2.031	*0.025*	2.31	*0.047*
	(0.586-2.959)		(1.093-3.774)		(1.011-5.277)	
cT stage	2.305	*0.013*	1.125	*0.647*	1.183	*0.618*
cT1-2 vs. cT3-4	(1.194-4.448)		(0.679-1.865)		(0.611-2.290)	
cN stage	1.661	*0.461*	0.937	*0.907*	1.49	*0.494*
cN0 vs. cN+	(0.431-6.402)		(0.317-2.773)		(0.475-4.675)	
cM stage (6th AJCC system)	0.788	*0.484*	0.764	*0.267*	0.742	*0.307*
cM0 vs. cM1a/b	(0.404-1.537)		(0.475-1.229)		(0.418-1.317)	
Lymph node dissection	2.438	*0.044*	1.333	*0.326*	1.16	*0.667*
2 fields vs 3 fields	(1.024-5.807)		(0.751-2.366)		(0.590-2.282)	
ypT stage	0.368	*0.016*	0.413	*0.004*	0.518	*0.094*
ypT0 vs. ypT1-4	(0.164-0.828)		(0.228-0.748)		(0.240-1.118)	
ypN stage	0.558	*0.167*	0.49	*0.02*	0.694	*0.322*
ypN0vs. ypN1-3	(0.244-1.276)		(0.269-0.892)		(0.336-1.431)	
p-complete response (pCR)	0.818	*0.772*	0.574	*0.228*	0.745	*0.6*
pCR vs. Non-pCR	(0.209-3.193)		(0.233-1.416)		(0.249-2.235)	
Adjuvant chemotherapy	0.824	*0.606*	1.479	*0.174*	1.51	*0.284*
Yes vs. no	(0.394-1.722)		(0.842-2.600)		(0.710-3.212)	
Surgical margin	0.176	*<0.001*	0.263	*<0.001*	0.187	*<0.001*
Negative vs. positive	(0.061-0.510)		(0.132-0.522)		(0.086-0.404)	

Abbreviations; HR, hazard ratio; CI, confidential interval

### Significance of supraclavicular and/or celiac LN metastasis

**Figure 3:**
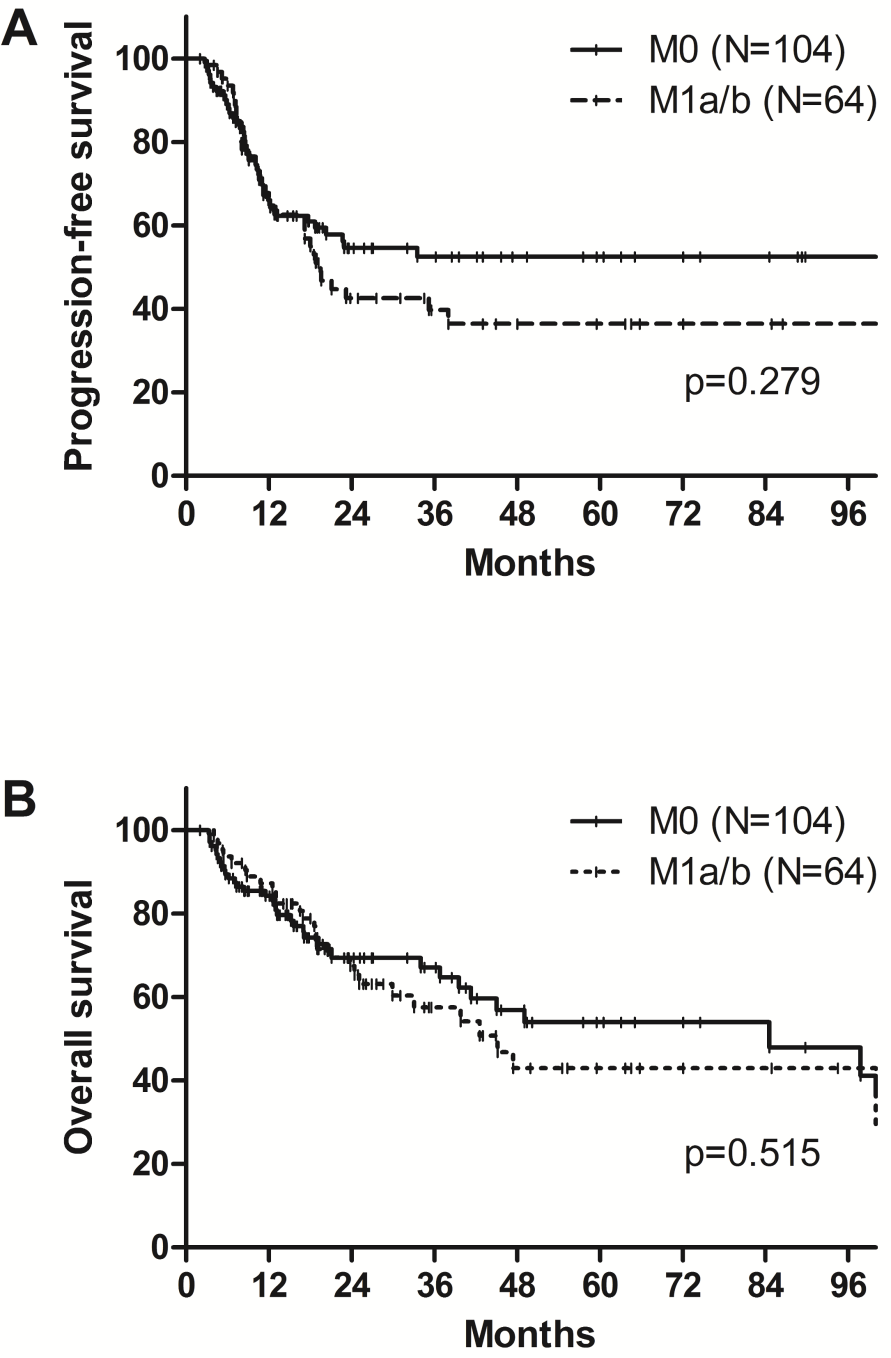
A. Progression-free survival and B. overall survival according to supraclavicular and/or celiac lymph node metastasis (cM1a/b) among patients who underwent surgery

Among all, the patients having metastasis to the supraclavicular and/or celiac LNs (cM1a/b group) showed numerically lower, but not significant, PFS (40.1% *vs*. 53.5%, *p* = 0.204) and OS (62.5% *vs*. 68.4%, *p* = 0.362) at 2 years, when compared to those having regional LN metastasis (cM0 group). Among 168 patients who underwent surgery, pCR rate was numerically higher in cM0 group, but not significant (29.8% *vs*. 20.3%, *p* = 0.174), and the same trends were shown on PFS (42.5% *vs*. 54.6%, *p* = 0.279) and OS (67.4% *vs*. 69.3%, *p* = 0.515) at 2 years (Figure 3).

## DISCUSSION

Supraclavicular and celiac LNs were defined as non-regional LNs of thoracic esophageal cancer in the 6^th^ AJCC staging system. However, a number of evidence had suggested that supraclavicular and/or celiac LN metastasis does not compromise prognosis compared to other regional LN metastasis [8-11]. Thus, in the 7^th^ AJCC staging system, discrimination of cervical and celiac LNs as non-regional LNs was abandoned. However, the prognostic impact of supraclavicular and/or celiac LN metastasis in the setting of NACRT and surgery has not been properly addressed yet, since most previous trials, including the CROSS trial, did not include the patients with cM1a/b stage by the 6^th^ AJCC staging system [3, 12, 13]. The current study revealed that the supraclavicular and/or celiac LN metastasis did not compromise survival outcomes in the patients who tolerated tri-modality treatment of NACRT and surgery.

Several prognostic factors have been suggested in esophageal cancer following NACRT and surgery such as tumor location, cT stage, surgical margin status, and pCR rate [14-17]. The present study also confirmed that negative resection margin was a significantly favorable prognostic factor for both PFS and OS in multivariate analysis (*p* < 0.001). pCR rate following NACRT is known as 25%~30% [3, 15, 16] though its prognostic significance is still controversial [18, 19]. In the current study, pCR was achieved in 26.2% and it was not a significant factor on OS at 2 year (79.8% *vs*. 64.3%, *p* = 0.118). This might be partly explained by the fact that OS at 2 years of patients with pT0N+ and pT+N0 (76.3% and 66.3%) were not different from that of patients achieving pCR (79.8%) (Figure 2).

Recently, the prognostic implication of FDG-PET parameters following NACRT was raised. Our institution previously reported the impact of FDG-PET parameter following NACRT in esophageal cancer patients [20]. In the present study, we could not incorporate the FDG-PET parameters into the prognostic factor analysis because the raw data of FDG-PET parameters were not available in 20% of the patients, whose FDG-PET CT was done at other hospitals. However, based on the available data in the previous study, SUVmax value reduction rates of 72.1% in main tumor and 50.7% in LNs estimated pCR with the best accuracy while total lesion glycolysis did not.

The clinical outcomes by the current study were favorably comparable with those by previous randomized studies, even though 37.7% of patients in the current study had M1a/b disease. The previous randomized studies reported OS rates of 60%~67 % at 2 years and 39%~47% at 5 years following NACRT and surgery [3, 12, 13]. In this study, the 2- and 5-year OS rates were 69.3% and 53.9%, respectively. Furthermore, the rates of R0 resection and postoperative mortality in hospital or within 30 days were 91.1% and 6.5%, respectively, which were also comparable with the previous studies (92.0% and 5.9% in the CROSS trial and 93.8% and 11.1% in the FFCD 9901 trial, respectively) [3, 13].

This study has a few limitations. First, this study included rather a limited number of patients to reach concrete conclusions. However, considering that we included only the patients with squamous cell carcinoma of the thoracic esophagus, who were treated in a highly homogenous manner, 199 patients is not a small number. Squamous cell carcinoma is the most prevalent histologic type in Asian countries such as China, Japan, and Korea. In fact, adenocarcinoma composed of only 2.5% of the patients who received NACRT for esophageal cancer in our institution. Second, a selection bias might have existed as NACRT and surgery was mainly recommended to those who had favorable performance status and were expected to tolerate large RT volume. In fact, chemotherapy alone before surgery was mainly recommended to the patients who had marginal performance status. Thus, our results might not be applicable to all patients having supraclavicular and/or celiac LN metastasis.

In summary, celiac and/or supraclavicular LN metastasis did not compromise the survival in patients with locally advanced esophageal squamous cell carcinoma who received NACRT and surgery. Further larger prospective studies should be warranted to establish the optimal treatment of thoracic esophageal cancer with celiac and/or cervical LN.

## MATERIALS AND METHODS

### Patients

NACRT and surgery was recommended as the definitive therapy to 201 patients for having locally advanced thoracic esophageal squamous cell carcinoma at the authors’ institute from January 2003 till July 2014. Following approval by our institutional review board, we retrospectively reviewed the medical records. After excluding two patients, who abandoned their treatment during NACRT course and were lost to our follow-up, analyses were done on 199. In all patients, endoscopy with biopsy, chest computed tomography (CT), and whole body 18F-fluorodeoxyglucose positron emission tomography-computed tomography (^18^F-FDG PET-CT) were performed for diagnosis and staging purpose. Endoscopic ultrasonography (EUS) was performed in 116 patients (58.3%). Primary tumor location was allocated according to the uppermost involvement of the primary tumor according to the AJCC 7^th^ edition.

### Treatment scheme

RT was to deliver 45 Gy in 25 fractions by 1.8 Gy per fraction over 5 weeks until March of 2009 in 54 patients (27.1%) and 44 Gy in 22 fractions by 2.0 Gy per fraction thereafter in 145 (72.9%). All patients received 3-dimensional conformal RT (3D-CRT), typically through 3 or 4 coplanar fields using 4-, 6-, or 10-MV photons from linear accelerator. Primary tumor and metastatic LNs were delineated as the gross tumor volume (GTV) based on CT, PET-CT, and endoscopic findings. The clinical target volume (CTV) of primary tumor included primary GTV plus 2~3 cm margins in the craniocaudal directions and 0.5 cm margin in the circumferential direction. The nodal CTV was delineated by putting 1 cm margin in all directions from the nodal GTV. Elective irradiation to the supraclavicular and/or celiac LNs was not employed in the current study. The planning target volume (PTV) was defined as 0.5~0.7 cm margin in all directions from the CTV to account for the respiratory motion and daily setup errors.

Two cycles of intravenous chemotherapy (5-flurouracil (5-FU) 1,000 mg/m^2^/day for 4 consecutive days plus cisplatin 60 mg/m^2^/day on the 1^st^ day) were administered at 3 weeks’ interval, and the 1^st^ cycle was to planned on the 1^st^ day of RT. Tumor response and resectability was re-evaluated before surgical resection by chest CT and FDG PET-CT in 3~4 weeks of NACRT completion.

Esophageal resection through thoracotomy was combined with either two-field or three-field lymphadenectomy. Two-field (thoracic and abdominal) LN dissection was performed mainly in the patients with lower thoracic esophageal cancer while three-field (cervical, thoracic, and abdominal) LN dissection was done mainly in those with upper or mid-thoracic esophageal cancer. Following NACRT, adjuvant chemotherapy or thoracic RT were optionally recommended to the patients with good performance status and risk factors based on surgical and pathological findings: 3 cycles of 5-FU and cisplatin chemotherapy was delivered in 36 patients (21.4%); 20 Gy in 10 fractions thoracic RT was in 13 (7.7%), respectively. Usual indications for adjuvant therapy included multiple pathologic lymph node metastases, close or positive resection margin, and/or difficulty of dissection experienced at time of surgery due to bulky primary tumor and/or metastatic LN.

### Statistical analyses

The durations of PFS and OS were calculated from the first date of NACRT till the date of any event of recurrence and death or the last follow-up, respectively. The distribution of categorical variables was analyzed by the chi-square test. The survival rates were calculated by the Kaplan-Meier method and were compared by log-rank test for univariate analysis. Multivariate analysis was described with HR and their 95% CI, derived from the cox proportional hazards model. Two-sided p-values of 0.05 or less were considered statistically significant. SPSS Statistics version 20 (SPSS Inc., an IBM Company, Chicago, IL) was used for the analysis.

## References

[1] Demeester SR (2009). Epidemiology and biology of esophageal cancer. Gastrointest Cancer Res.

[2] Allum WH, Stenning SP, Bancewicz J, Clark PI, Langley RE (2009). Long-term results of a randomized trial of surgery with or without preoperative chemotherapy in esophageal cancer. J Clin Oncol.

[3] van Hagen P, Hulshof MC, van Lanschot JJ, Steyerberg EW, van Berge Henegouwen MI, Wijnhoven BP, Richel DJ, Nieuwenhuijzen GA, Hospers GA, Bonenkamp JJ, Cuesta MA, Blaisse RJ, Busch OR (2012). Preoperative chemoradiotherapy for esophageal or junctional cancer. N Engl J Med.

[4] Sjoquist KM, Burmeister BH, Smithers BM, Zalcberg JR, Simes RJ, Barbour A, Gebski V, Australasian Gastro-Intestinal Trials G (2011). Survival after neoadjuvant chemotherapy or chemoradiotherapy for resectable oesophageal carcinoma: an updated meta-analysis. Lancet Oncol.

[5] Fan M, Lin Y, Pan J, Yan W, Dai L, Shen L, Chen K (2016). Survival after neoadjuvant chemotherapy *versus* neoadjuvant chemoradiotherapy for resectable esophageal carcinoma: A meta-analysis. Thorac Cancer.

[6] Frederick LG, Page DL, Fleming ID, Fritz AG, Balch CM, Haller DG, Morrow M (2002). AJCC Cancer Staging Manual.

[7] Edge S, Byrd DR, Compton CC, Fritz AG, Greene FL, Trotti A (2010). AJCC Cancer Staging Manual. New York: Springer-Verlag.

[8] Lee PC, Port JL, Paul S, Stiles BM, Altorki NK (2009). Predictors of long-term survival after resection of esophageal carcinoma with nonregional nodal metastases. Ann Thorac Surg.

[9] Lin CS, Chang SC, Wei YH, Chou TY, Wu YC, Lin HC, Wang LS, Hsu WH (2009). Prognostic variables in thoracic esophageal squamous cell carcinoma. Ann Thorac Surg.

[10] Tachimori Y, Ozawa S, Numasaki H, Matsubara H, Shinoda M, Toh Y, Udagawa H (2014). Supraclavicular node metastasis from thoracic esophageal carcinoma: A surgical series from a Japanese multi-institutional nationwide registry of esophageal cancer. J Thorac Cardiovasc Surg.

[11] Rizk N, Venkatraman E, Park B, Flores R, Bains MS, Rusch V, American Joint Committee on Cancer staging s (2006). The prognostic importance of the number of involved lymph nodes in esophageal cancer: implications for revisions of the American Joint Committee on Cancer staging system. J Thorac Cardiovasc Surg.

[12] Tepper J, Krasna MJ, Niedzwiecki D, Hollis D, Reed CE, Goldberg R, Kiel K, Willett C, Sugarbaker D, Mayer R (2008). Phase III trial of trimodality therapy with cisplatin, fluorouracil, radiotherapy, and surgery compared with surgery alone for esophageal cancer: CALGB 9781. J Clin Oncol.

[13] Mariette C, Dahan L, Mornex F, Maillard E, Thomas PA, Meunier B, Boige V, Pezet D, Robb WB, Le Brun-Ly V, Bosset JF, Mabrut JY, Triboulet JP, Bedenne L, Seitz JF (2014). Surgery alone *versus* chemoradiotherapy followed by surgery for stage I and II esophageal cancer: final analysis of randomized controlled phase III trial FFCD 9901. J Clin Oncol.

[14] Kaklamanos IG, Walker GR, Ferry K, Franceschi D, Livingstone AS (2003). Neoadjuvant treatment for resectable cancer of the esophagus and the gastroesophageal junction: a meta-analysis of randomized clinical trials. Ann Surg Oncol.

[15] Chirieac LR, Swisher SG, Ajani JA, Komaki RR, Correa AM, Morris JS, Roth JA, Rashid A, Hamilton SR, Wu TT (2005). Posttherapy pathologic stage predicts survival in patients with esophageal carcinoma receiving preoperative chemoradiation. Cancer.

[16] Donahue JM, Nichols FC, Li Z, Schomas DA, Allen MS, Cassivi SD, Jatoi A, Miller RC, Wigle DA, Shen KR, Deschamps C (2009). Complete pathologic response after neoadjuvant chemoradiotherapy for esophageal cancer is associated with enhanced survival. Ann Thorac Surg.

[17] Wang BY, Liu CY, Lin CH, Hsu PK, Hsu WH, Wu YC, Cheng CY (2012). Endoscopic tumor length is an independent prognostic factor in esophageal squamous cell carcinoma. Ann Surg Oncol.

[18] Berger AC, Farma J, Scott WJ, Freedman G, Weiner L, Cheng JD, Wang H, Goldberg M (2005). Complete response to neoadjuvant chemoradiotherapy in esophageal carcinoma is associated with significantly improved survival. J Clin Oncol.

[19] Wang CC, Cheng JC, Tsai CL, Lee JM, Huang PM, Lin CC, Hsu CH, Hsieh MS, Chang YL, Hsu FM (2015). Pathological stage after neoadjuvant chemoradiation and esophagectomy superiorly predicts survival in patients with esophageal squamous cell carcinoma. Radiother Oncol.

[20] Park JS, Choi JY, Moon SH, Ahn YC, Lee J, Kim D, Kim K, Shim YM (2013). Response evaluation after neoadjuvant chemoradiation by positron emission tomography-computed tomography for esophageal squamous cell carcinoma. Cancer Res Treat.

